# Human Exposure to Heavy Metals over the Last 100 Years

**DOI:** 10.1007/s00128-024-03933-7

**Published:** 2024-08-02

**Authors:** Kamil Brudecki, Agnieszka Pasieka, Renata Franczak, Anna Pankowska, Małgorzata Kołodziej, Jadwiga Lorenc-Brudecka, Edyta Łokas

**Affiliations:** 1 Institute of Nuclear Physics, Polish Academy of Sciences, Radzikowskiego 152, Kraków, 31-342 Poland; 2 Nature Education Centre of the Jagiellonian University, Gronostajowa 5, Kraków, 30-387 Poland

**Keywords:** Heavy Metals, Internal Exposure, Mass Spectrometry, Human

## Abstract

The primary objective of the presented research was to assess the impact of intense global economic development, over the last 100 years, on the bioaccumulation of heavy metals in the human body. This evaluation was conducted based on the measurement of heavy metals in human hair samples collected 100 years ago in present-day southern Poland.In this study, concentrations of lead (Pb), cadmium (Cd), arsenic (As), zinc (Zn), copper (Cu), iron (Fe), and selenium (Se) were measured in 61 hair samples,28 of which were obtained from individuals who lived 100 years ago, while the remaining 33 constituted the contemporary control group. The concentrations were determined using a triple quadrupole inductively coupled plasma mass spectrometer (Agilent 8900). Statistical analysis of the obtained results was conducted using the Principal Cmponent Analysis and the non-parametric Mann-Whitney test. In the case of As, Pb, Cd, and Fe, the concentrations were significantly higher in individuals who lived 100 years ago compared to those living today. Over this period, the median concentrations were shown to have decreased by 95%, 94%, 85%, and 69% for As, Pb, Cd, and Fe, respectively. No statistically significant differences were observed for Cu, Zn, and Se. The results obtained for Pb, Cd, As, and Fe unequivocally indicate that the population studied from 100 years ago was more exposed to internal contamination with these metals than people who live nowadays.

## Introduction

Human biomonitoring is applied to evaluate exposure of a given population to chemical elements from anthropogenic and natural sources. The level of elements in hair is related to the regional exposure, as well as to the environmental and hygienic factors. Hair analysis is useful for identification of a population that is environmentally exposed (Gil and Hernández 2015; González-Muñoz et al. 2008; Nowak 1998), and usually reflects the exposure during the past 4–8 weeks. Hair can also be used to evaluate deficiency of essential elements as an indicator of metabolic and physiological disorders, like mental disorders, diabetes, anemia or rheumatoid arthritis (Nakaona et al. 2020).

The heavy metals are a group of chemical elements without a strict definition. From a chemical-physical perspective, heavy metals are characterised by high density and atomic weight, and primarily include lead (Pb), cadmium (Cd), mercury (Hg), arsenic (As), chromium (Cr), nickel (Ni), copper (Cu), and zinc (Zn). However, from a biological standpoint, the fundamental trait of heavy metals is their high toxicity to living organisms. In this context, the list mentioned above can be extended by including aluminium (Al) and selenium (Se).

Sources of heavy metals in the environment have both natural and anthropogenic origins. Among the main anthropogenic sources of heavy metals in the natural environment are heavy industry (metallurgical, chemical, or refinery), the combustion of fossil fuels (power plants, heating plants, automotive transport), and intensive agriculture (artificial fertilisers, plant protection agents) (Briffa et al. 2020; Callender 2003; Tchounwou et al. 2012).

Heavy metals primarily enter the human body through both the digestive and respiratory pathways, with a tendency to accumulate in the nervous system. The consequences of heavy-metal poisoning in the case of high doses over a short period are well-known to science. However, the health effects of long-term exposure to heavy metals in small doses are relatively poorly understood and ambiguous. It is suspected that heavy metals may influence the onset of neurological diseases (Alzheimer’s, Parkinson’s, multiple sclerosis), cancers (respiratory system tumours), hormonal disorders, impairment of the immune system, allergies, and reproductive and developmental diseases (fertility issues, miscarriages, congenital defects, and delayed physical and cognitive development in children)(Adetutu et al. 2023; Briffa et al. 2020; Fu and Xi 2020; Jaishankar et al. 2014; Rehman et al. 2018; Sodhi et al. 2022; Song and Li 2015; Tchounwou et al. 2012; Witkowska et al. 2021; Vardhan et al. 2019).

The primary objective of the presented research was to assess bioaccumulation of heavy metals in the human body over the last 100 years This evaluation was conducted based on the measurement of heavy metals in human hair samples collected 100 years ago in present-day southern Poland. Subsequently, the results were compared with those of contemporary individuals. The research will indirectly provide information about the emission of heavy metals into the environment and the associated air pollution from 100 years ago. Furthermore, the presented research will provide information on the direction and dynamics of changes in environmental contamination with heavy metals.

## Materials and Methods

### Samples

The most significant research material used in the presented publication consists of samples of human hair obtained from individuals living 100 years ago in the area of present-day southern Poland. These samples belong to the Natural Education Centre of the Jagiellonian University in Krakow, and originate from the collection of Professor Julian Talko-Hryncewicz (1850–1936).Professor Julian Talko-Hryncewicz was a distinguished anthropologist, ethnographer, physician, traveller, and professor at the Jagiellonian University and the University of Vilnius. He conducted extensive anthropological studies. In the Natural Education Centre’s collection, several hundred samples of human hair have been preserved from people contemporary to him, originating both from Poland and from around the world. Hair samples were stored in glass frames without any added chemicals. Due to their age, these samples are of great value to us. Additionally, documentation related to the collection has been preserved, and contains information about the nationality, residence, age, and gender of the individuals from whom the hair samples were taken.

The research involved 28 samples of human hair (12 from men; 16 from women) from over 100 years ago. The individuals from whom the samples were taken lived in the area of present-day southern Poland. At the time of sampling, they ranged in age from 9 to 54 years old. The control group consisted of 33 hair samples taken from individuals currently live in the southern region of Poland (the city of Krakow and its surroundings). The control group included 22 men and 11 women, with the age of 3 to 67 years. Detailed information about the study participants are presented in Table 1. The hair of individuals in the control group was not dyed. The research obtained the approval from the Bioethics Committee at the Regional Medical Chamber in Krakow (decision number 173/KBL/OIL/2023, date 06.07.2023).

**Table 1:** Detailed information about the study and control group

Sample Code	Type	Sex	Age	Location	Sample Code	Type	Sex	Age	Location
S1	STUDY	M	9	Kielce(Poland)	C4	CONTROL	F	5	Kraków(Poland)
S2	STUDY	M	9	Kielce(Poland)	C5	CONTROL	M	5	Kraków(Poland)
S3	STUDY	M	10	Kielce(Poland)	C6	CONTROL	F	7	Kraków(Poland)
S4	STUDY	M	10	Kielce(Poland)	C7	CONTROL	M	8	Kraków(Poland)
S5	STUDY	M	10	Kielce(Poland)	C8	CONTROL	F	11	Kraków(Poland)
S6	STUDY	M	11	Kielce(Poland)	C9	CONTROL	M	12	Kraków(Poland)
S7	STUDY	M	11	Kielce(Poland)	C10	CONTROL	M	13	Kraków(Poland)
S8	STUDY	M	11	Kielce(Poland)	C11	CONTROL	M	14	Kraków(Poland)
S9	STUDY	M	12	Kielce(Poland)	C12	CONTROL	M	14	Kraków(Poland)
S10	STUDY	M	12	Kielce(Poland)	C13	CONTROL	F	14	Kraków(Poland)
S11	STUDY	M	12	Kielce(Poland)	C14	CONTROL	F	22	Kraków(Poland)
S12	STUDY	F	13	Kielce(Poland)	C15	CONTROL	M	22	Kraków(Poland)
S13	STUDY	M	13	Kielce(Poland)	C16	CONTROL	F	26	Kraków(Poland)
S14	STUDY	F	18	Brzezna(Poland)	C17	CONTROL	M	27	Kraków(Poland)
S15	STUDY	F	18	Brzezna(Poland)	C18	CONTROL	M	27	Kraków(Poland)
S16	STUDY	F	21	Brzezna(Poland)	C19	CONTROL	M	29	Kraków(Poland)
S17	STUDY	F	24	Brzezna(Poland)	C20	CONTROL	F	32	Kraków(Poland)
S18	STUDY	F	27	Brzezna(Poland)	C21	CONTROL	F	32	Kraków(Poland)
S19	STUDY	F	30	Brzezna(Poland)	C22	CONTROL	M	34	Kraków(Poland)
S20	STUDY	F	34	Brzezna(Poland)	C23	CONTROL	M	35	Kraków(Poland)
S21	STUDY	F	34	Brzezna(Poland)	C24	CONTROL	F	36	Kraków(Poland)
S22	STUDY	F	35	Brzezna(Poland)	C25	CONTROL	F	37	Kraków(Poland)
S23	STUDY	F	37	Brzezna(Poland)	C26	CONTROL	F	39	Kraków(Poland)
S24	STUDY	F	37	Brzezna(Poland)	C27	CONTROL	M	39	Kraków(Poland)
S25	STUDY	F	38	Brzezna(Poland)	C28	CONTROL	M	40	Kraków(Poland)
S26	STUDY	F	42	Brzezna(Poland)	C29	CONTROL	M	43	Kraków(Poland)
S27	STUDY	F	52	Brzezna(Poland)	C30	CONTROL	M	60	Kraków(Poland)
S28	STUDY	F	54	Brzezna(Poland)	C31	CONTROL	M	62	Kraków(Poland)
C1	CONTROL	M	3	Kraków(Poland)	C32	CONTROL	M	66	Kraków(Poland)
C2	CONTROL	M	3	Kraków(Poland)	C33	CONTROL	M	67	Kraków(Poland)
C3	CONTROL	M	5	Kraków(Poland)					

### Sample Preparation and Mineralisation

The hair samples from participants were cut from the nape of the neck.The weight of hair samples prepared for mass spectrometry measurements was usually less than 0.5 g (grams). The hair samples were cleaned to have any contamination from the outer surfaces removed, by serially washing with solvents according to the following scheme: acetone (10 min); ultra-pure water (10 min); ultra-pure water (10 min); ultra-pure water (10 min); acetone (10 min); and, finally ultra-pure water (10 min) (Morton et al. 2002; Druyan et al. 1998; Pozebon et al. 2017). The electrical resistance of the ultra-pure water was at the level of 18·10^4^ ohm-meters, while total organic carbon fractions was at the level of 3 part per billion. Purity of the acetone was at the level of 99.9%. Next, the cleaned samples were mineralised in a microwave digestion system (PreeKem) using 3 millilitres of 65% ultra-pure nitric acid (Merck). Mineralization proceeded in two stages. In the first stage, the sample was heated to a temperature of 140 °C(ramp time 8 min, hold time 2 min). Then, the sample was heated to a temperature of 180 °C(ramp time 10 min, hold time 10 min). After mineralisation, the samples were diluted to a 2% nitric acid matrix using ultra-pure water (Merck Milli-Q) and transferred into the mass spectrometer.

### Measurements

The concentrations of seven elements (Pb, Cd, As, Zn, Cu, Fe, and Se) of human hair samples were determined by using a triple quadrupole inductively coupled plasma mass spectrometer (Agilent 8900). The spectrometer was operated in collision-reaction mode, with a helium flow of 4.5 millilitres per minute and in dual quad mode. Calibration was performed with using calibration solutions of the selected elements specifically designed for mass spectrometry (Carl Roth). Spectrometer calibration involved a minimum of 6 points. To achieve optimal measurement precision, the selected elements in the sample were measured at least 100 times. The entire measurement process for one sample took approximately 4 min.^115^I was used as an internal standard. The exact operating parameters of the spectrometer are described in Table 2.

**Table 2:** Mass spectrometer operating conditions

Acquisition mode	MS/MS (dual mode)
**RF power [W]**	1550
**Sampling depth [mm]**	8
**Gas flow rate [L/min]**	1.07
**Make-up gas flow rate [L/min]**	0
**Spray chamber temperature [°C]**	2
**He cell gas flow rate [mL/min]**	4.5
**Internal standard isotope**	^115^In

Typical detection limits were 0.04 ng/L (nanogram per liter), 0.001 ng/L, 0.004 ng/L, 3 ng/L, 0.9 ng/L, 0.9 ng/L and 0.5 ng/L respectively for Pb, Cd, As, Zn, Cu, Fe, and Se. In the case of Fe, Zn, Cu, and Pb, all results were above the detection limits. For As and Cd, the majority of the obtained results (95%) were above the detection limits, while, for Se, only 36% of the results were above its detection limit.

Quality control was conducted by using two reference materials of human hair, namely ERM-DB001 (Institute for Reference Materials and Measurements, European Commission’s Joint Research Centre) and IAEA-086 (Analytical Quality Control Services, International Atomic Energy Agency). Measurements of the reference material and the blank (2% nitric acid) were performed every 15 samples. In total, measurements were carried out for 10 samples of reference materials. This comprehensive analytical approach aims to ensure accuracy and reliability in the determination of trace metal concentrations in the samples under investigation.

The accuracy of the results was confirmed through measurements of reference materials. The obtained results align with the certified values. The highest agreement was achieved for As and Cu, with values of 100 ± 5% and 100 ± 6%, respectively. The lowest agreement was observed for Pb, at 105 ± 2%. Detailed results of the determinations for reference materials are presented in Table 3.

**Table 3:** Mass spectrometry quality assurance

Referencesmatherial	Element	Certifiedvalue [mg kg^− 1^]	Numer of measurments	Average obtained value [mg kg^− 1^]	Recovery [%]
ERM – DB001	As	0.044 ± 0.006	5	0.044 ± 0.002	100 ± 5
	Cd	0.125 ± 0.007	5	0.128 ± 0.005	102 ± 4
	Cu	33 ± 4	5	33 ± 2	100 ± 6
	Pb	2.14 ± 0.20	5	2.25 ± 0.05	105 ± 2
	Se	3.24 ± 0.24	5	3.27 ± 0.12	101 ± 4
	Zn	209 ± 12	5	218 ± 7	104 ± 3
IAEA 086	Fe	123 ± 13	5	121 ± 5	98 ± 4
	Zn	167 ± 8	5	172 ± 5	103 ± 3
	Cu	17.6 ± 1	5	18.0 ± 0.9	102 ± 5
	Se	1 ± 0.2	5	1 ± 0.1	100 ± 10

### Statistical Analysis

The statistical analysis was conducted in two stages. In the first stage, correlations between the examined metal concentrations were assessed using Principal Component Analysis (PCA) method. Next, differences between the study group and the control group were evaluated using the non-parametric Mann-Whitney test.

**Fig. 1:**
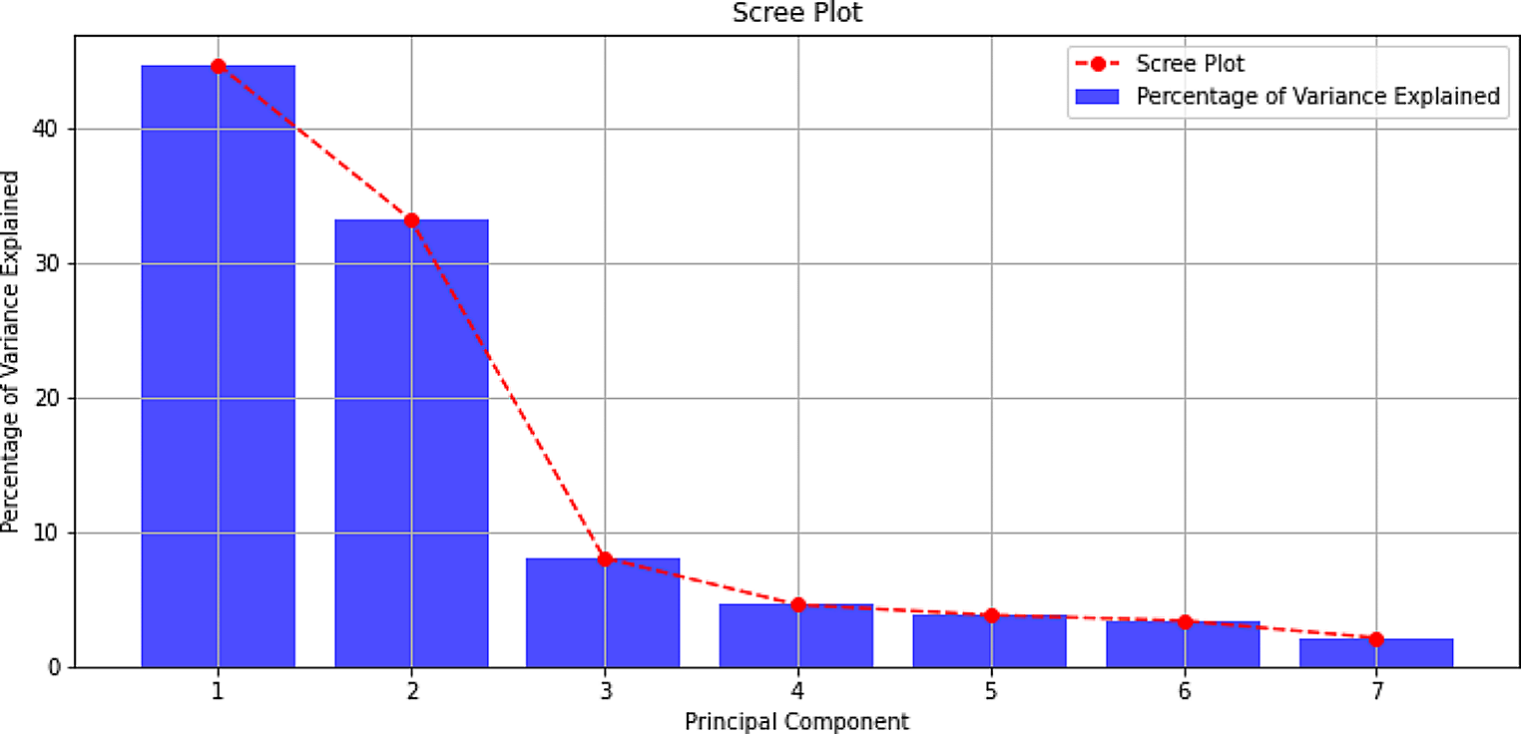
Percentage of variance explained for principial components

PCA method is a statistical technique used in data exploration. Its main objective is to simplify complex data sets by reducing dimensionality while retaining as much original data variability as possible. In the presented analysis, the concentrations of seven studied heavy metals in both groups were analysed. The first step of the analysis was to calculate the covariance matrix to identify the covariances between variables. Next, eigenvalues and their corresponding eigenvectors were determined based on the covariance matrix. The obtained eigenvalues are presented in Table 4. Additionally, a variance visualization (Scree plot) was performed. The explained percentage of variance is 44.6%, 33.2%, 8.1%, 4.6%, 3.9%, 3.4%, and 2.2% for PC1 (principal components), PC2, PC3, PC4, PC5, PC6, and PC7, respectively. The explained percentage of variance are presented in Fig. 1. Finally, based on the scree plot and eigenvalue analysis, two principal components were selected, which explain 77.8% of the total variance. Calculations were performed with using the RStudio program (Posit PBC, USA).

**Table 4:** Eigenvalues for principal components (PC) and cumulative variance explained

Principal Component (PC)	Eigenvalue	Cumulative Variance [%]
PC1	3.18	44.6
PC2	2.36	77.8
PC3	0.58	85.9
PC4	0.33	90.5
PC5	0.27	94.4
PC6	0.24	97.8
PC7	0.15	100

In additions to assess the significance of differences in heavy metal content between the individual groups, the non-parametric Mann-Whitney test was used (data did not meet the assumptions of normality). Calculations were performed with using the RStudio program (Posit PBC, USA).

The Shapiro-Wilk test was performed to check if the data distribution is normal. The results of the Shapiro-Wilk test indicated that the data distribution is not-normal. Therefore, non-parametric methods were applied for data analysis, which does not require the assumption of normality of the data distribution. Median was used to present the data because it is a measure of central tendency that better reflects asymmetric distributions than the mean. To compare the two groups, the non-parametric Mann-Whitney test was used, which is the equivalent of the t-test for independent samples but does not require the assumption of normality of the data distribution.

The Mann-Whitney test, being a non-parametric method, is robust to deviations from normality and is suitable for comparing distributions of two independent groups. Its application in this context allows for a reliable evaluation of whether there are statistically significant differences in the concentrations of heavy metals between the historical hair samples and the contemporary control group. The use of RStudio ensures a rigorous and standardised statistical analysis of the obtained data, enhancing the reliability of the study’s conclusions.

## Results and Discussion

In this study, concentrations of lead (Pb), cadmium (Cd), arsenic (As), zinc (Zn), copper (Cu), iron (Fe), and selenium (Se) were measured among 61 hair samples, 28 of which were obtained from individuals who lived 100 years ago, while the remaining 33 constituted the contemporary control group.

**Fig. 2:**
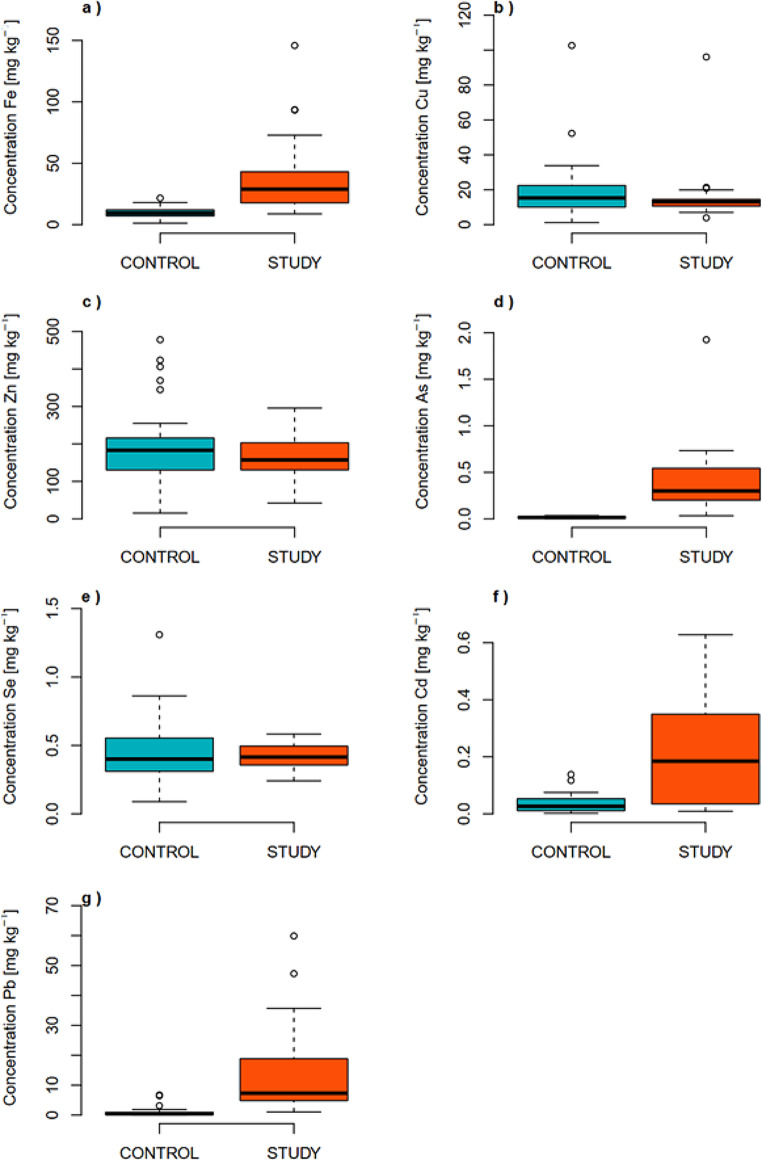
Comparison of concentration results in the control and study groups for Fe (**a**), Cu (**b**), Zn (**c**), As (**d**), Se (**e**), Cd (**f**) and Pb (**g**)

In the case of As, Pb, Cd and Fe, the concentrations were higher in individuals who lived 100 years ago compared to those living today. In the studied group, the median concentrations were 0.30 ± 0.13 mg/kg (milligram per kilogram), 7.76 ± 0.66 mg/kg, 0.18 ± 0.10 mg/kg, and 28.9 ± 1.2 mg/kg for As, Pb, Cd, and Fe, respectively. For the control group, these values were 0.0150 ± 0.0020 mg/kg, 0.450 ± 0.019 mg/kg, 0.0265 ± 0.0054 mg/kg, and 9.38 ± 0.66 mg/kg, respectively. In the case of Cu, Zn and Se the concentrations were similar in individuals who lived 100 years ago compared to those living today. The median concentration for Cu in the study group was 13.1 ± 0.8 mg/kg, while in the control group, it was 15.4 ± 1.1 mg/kg. For Zn, the median in the study group was 157.3 ± 3.1 mg/kg, and in the control group, it was 180 ± 10 mg/kg. Meanwhile, for Se, the median concentration in the study group was 0.415 ± 0.052 mg/kg and in the control group 0.382 ± 0.024 mg/kg. Uncertainties were calculated according to uncertainty propagation law and they are at the level one sigma. However, it should be noted that not all heavy metals tend to accumulate in hair. For example, copper, even in extreme cases such as Wilson’s disease, does not tend to deposit in hair (Watt et al. 1995). The exact concentration results are presented in Table 5; Fig. 2.

**Table 5:** Heavy metals concentration results in study and control group

Element		Concentrations [mg kg^− 1^]				
		Minimum	1st Quartile	Median	3rd Quartile	Maximum
Fe	Control	1.173 ± 0.019	7.07 ± 0.19	9.38 ± 0.66	12.05 ± 0.55	21.6 ± 1.2
	Study	8.79 ± 0.43	18.65 ± 0.87	28.9 ± 1.2	42.8 ± 1.5	145.9 ± 3.4
Cu	Control	1.204 ± 0.038	10.07 ± 0.38	15.4 ± 1.1	22.86 ± 0.57	102.6 ± 5.6
	Study	3.924 ± 0.082	10.63 ± 0.27	13.06 ± 0.84	14.44 ± 0.35	96.1 ± 2.6
Zn	Control	15.58 ± 0.69	130.4 ± 5.5	180 ± 10	212.6 ± 8.5	480 ± 30
	Study	42.3 ± 1.3	130.8 ± 3.2	157.3 ± 3.1	201.4 ± 3.8	300 ± 10
As	Control	0.0040 ± 0.0010	0.009 ± 0.001	0.0150 ± 0.0020	0.021 ± 0.003	0.0350 ± 0.0050
	Study	0.0033 ± 0.0030	0.20 ± 0.13	0.30 ± 0.13	0.53 ± 0.18	1.925 ± 0.060
Se	Control	0.089 ± 0.014	0.31 ± 0.27	0.382 ± 0.024	0.55 ± 0.33	1.31 ± 0.17
	Study	0.241 ± 0.023	0.36 ± 0.32	0.415 ± 0.052	0.50 ± 0.31	0.583 ± 0.049
Cd	Control	0.004 ± 0.0010	0.0118 ± 0.0054	0.0265 ± 0.0054	0.0530 ± 0.0060	0.1380 ± 0.0090
	Study	0.0090 ± 0.0010	0.0373 ± 0.0054	0.18 ± 0.10	0.34 ± 0.14	0.628 ± 0.016
Pb	Control	0.053 ± 0.002	0.207 ± 0.006	0.450 ± 0.019	0.953 ± 0.022	6.82 ± 0.36
	Study	1.131 ± 0.023	5.18 ± 0.49	7.76 ± 0.66	17.9 ± 1.2	61.4 ± 1.5

The PCA method showed that the first principal component (PC1) explained 44.6% of the variance and was correlated with the concentrations of Fe, Ar, Cd, and Pb. The second principal component (PC2) explained 33.2% of the variance and was correlated with the concentrations of Se, Zn, and Cu. The PCA loadings table is shown in Table 6. Next, the data were transformed into a new two – dimensional space using the selected principal components.

**Table 6:** Loadings for the significant principal components (PC1 and PC2)

Element	Principal Component Loading	
	PC1	PC2
Fe	0.88	0.15
Cu	0.01	0.90
Zn	-0.26	0.86
Se	-0.29	0.85
As	0.75	-0.01
Cd	0.92	0.20
Pb	0.91	0.17

**Fig. 3:**
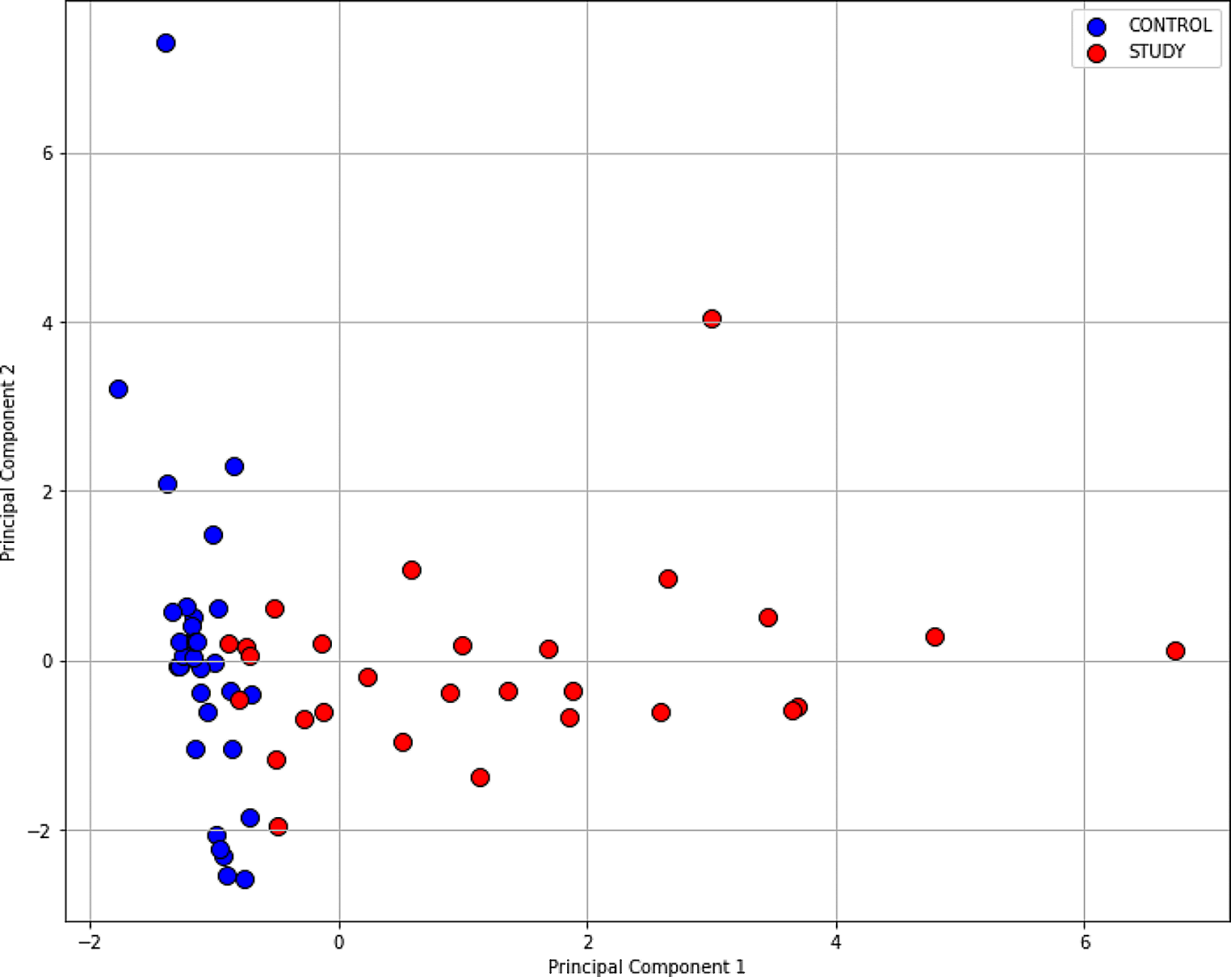
Principal Component Analysis (PCA) biplot

Figure 3 presents the data visualization in a dimensional space. The next step was to perform the Mann-Whitney test for the PC1 and PC2 values in the study group and the control group. The Mann-Whitney test confirmed the statistical significance differences for the PC1 values (p – value 8.52 × 10^–11^) and refuted the statistical significance differences for the PC2 values (p – value 0.59).

In summary, the statistical analysis revealed that the concentrations of As, Pb, Cd, and Fe are correlated, and that these concentrations were significantly higher in individuals from 100 years ago compared to those living today. On the other hand, although the concentrations of Se, Zn, and Cu are correlated, there are no statistically significant differences between the study and control groups.

The results obtained for Pb, Cd, As, and Fe unequivocally indicate that the population studied from 100 years ago was more exposed to internal contamination with these metals than people currently living. Over the past 100 years, the median concentrations decreased by 95%, 94%, 85%, and 69% for As, Pb, Cd, and Fe, respectively. The presented results align with the observed trend of intensive reduction in heavy metal emissions into the environment. Between 2005 and 2021, the European Union reduced lead (Pb) emissions into the natural environment by 42% and cadmium (Cd) by 37% (EEA 2023). In the United States, from 1970 to 1995, lead emissions into the atmosphere were reduced by 98% (Callender 2003). This also translates into reduced levels of heavy metal accumulation in the human body. For example, over the last 40 years, the average concentration of lead in the blood of U.S. residents has decreased by 96%, from 12.8 µg/dL (micrograms per deciliter) to 0.82 µg/dL (Dignam et al. 2019).

The results obtained for the control group are consistent with the literature data, indirectly confirming the correctness of the results (Drobyshev et al. 2017; González-Muñoz et al. 2008; Nowak 1998; Nowak and Chmielecka 2000; Wanget al. 2009).

**Fig. 4:**
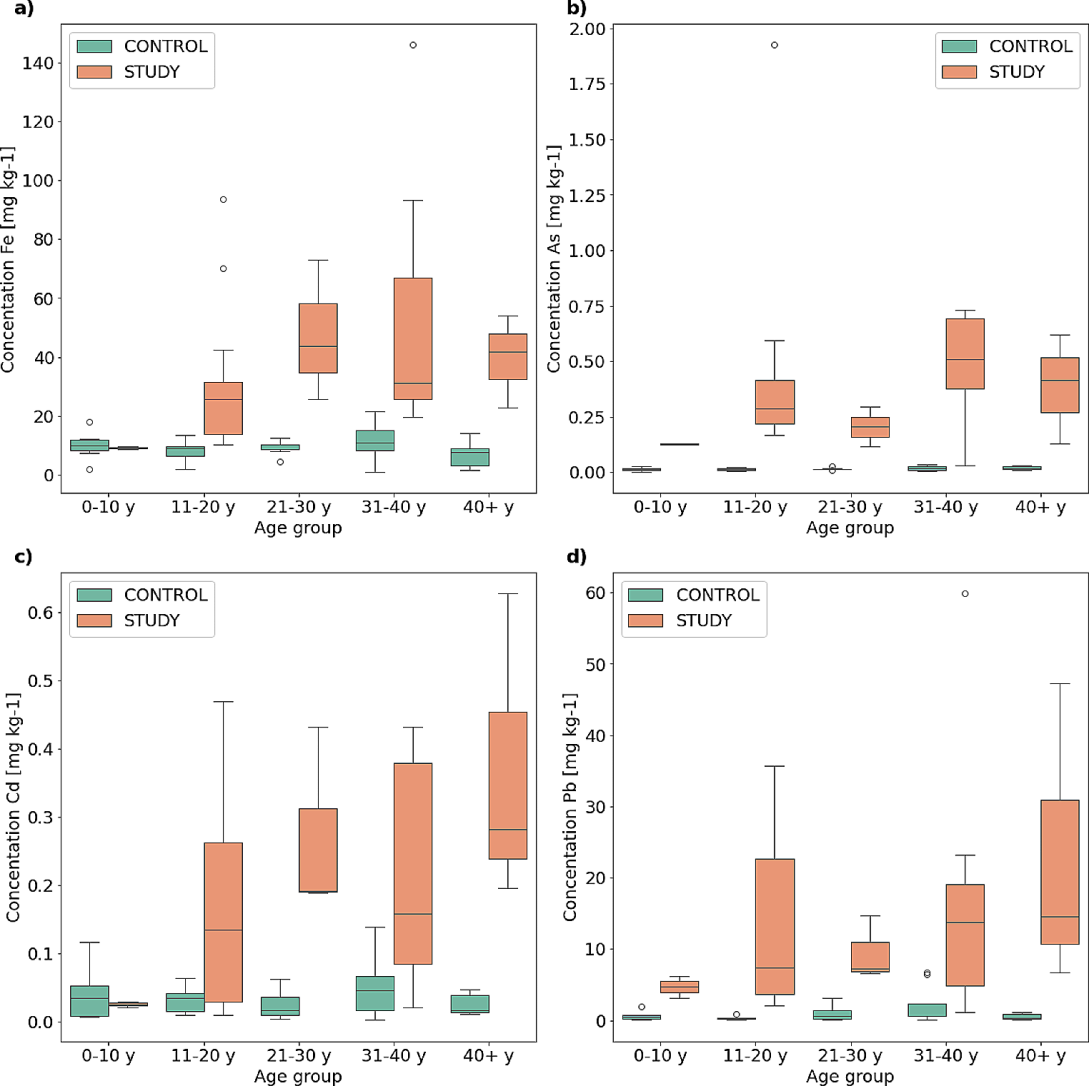
Comparison of concentration results in the control and study groups dividing them into age groups for Fe (**a**), As (**b**), Se (**e**), Cd (**c**) and Pb (**d**)

In the case of As, Pb, Cd, and Fe, results were analysed dividing them into age groups with 10-year intervals. The analysis results are presented in Fig. 4. For Pb and As, the concentrations measured in all of age groups are significantly higher in the individuals from 100 years ago. For Fe and Cd, there is no difference in concentrations in the first age group (up to 10 years), but in the remaining groups, the concentrations measured in the individuals who lived 100 years ago are also significantly higher. However, the analysis should be treated with caution due to the small study group size.

Despite the presented results pertaining to the population residing in today’s southern Poland, the conclusions drawn from them should be applicable to all Europeans and residents of the USA. Both in Europe and the USA, the processes of industrialization and subsequent environmental protection unfolded very similarly.

Although the key samples date back 100 years, and little can be said about the methodology of their collection, it is rather unlikely that they were contaminated. Firstly, the samples were carefully washed before measurements. Secondly, the hair was stored without the use of any chemical substances.

## Conclusions

Conducted studies have clearly indicated that people living 100 years ago were much more exposed to internal contamination by heavy metals such as As, Pb, Cd, and Fe compared to people living today. Over the past 100 years, numerous regulations have been introduced to improve air quality, protect water sources, and preserve ecosystems. Public awareness of the need to protect the natural environment and pursue sustainable development has also increased. Additionally, many new technologies have been developed over the past century, such as nuclear energy, renewable energy sources, and environmentally friendly transportation. All these factors have contributed to a significant reduction in the emission of heavy metals into the environment during this period, leading to a concurrent decrease in the internal contamination of the human body by these metals.
